# Development and Validation of an Automated Image-Based Deep Learning Platform for Sarcopenia Assessment in Head and Neck Cancer

**DOI:** 10.1001/jamanetworkopen.2023.28280

**Published:** 2023-08-10

**Authors:** Zezhong Ye, Anurag Saraf, Yashwanth Ravipati, Frank Hoebers, Paul J. Catalano, Yining Zha, Anna Zapaishchykova, Jirapat Likitlersuang, Christian Guthier, Roy B. Tishler, Jonathan D. Schoenfeld, Danielle N. Margalit, Robert I. Haddad, Raymond H. Mak, Mohamed Naser, Kareem A. Wahid, Jaakko Sahlsten, Joel Jaskari, Kimmo Kaski, Antti A. Mäkitie, Clifton D. Fuller, Hugo J. W. L. Aerts, Benjamin H. Kann

**Affiliations:** 1 Artificial Intelligence in Medicine Program, Mass General Brigham, Harvard Medical School, Boston, Massachusetts; 2 Department of Radiation Oncology, Dana-Farber Cancer Institute and Brigham and Women’s Hospital, Harvard Medical School, Boston, Massachusetts; 3 Department of Radiation Oncology (Maastro), GROW School for Oncology and Reproduction, Maastricht University Medical Center+, Maastricht, the Netherlands; 4 Department of Biostatistics, Harvard T.H. Chan School of Public Health, Boston, Massachusetts; 5 Department of Data Science, Dana-Farber Cancer Institute, Harvard Medical School, Boston, Massachusetts; 6 Radiology and Nuclear Medicine, CARIM and GROW, Maastricht University, Maastricht, the Netherlands; 7 Department of Medical Oncology, Dana-Farber Cancer Institute, Harvard Medical School, Boston, Massachusetts; 8 Department of Radiation Oncology, The University of Texas MD Anderson Cancer Center, Houston, Texas; 9 Department of Computer Science, Aalto University School of Science, Espoo, Finland; 10 Department Otorhinolaryngology–Head and Neck Surgery, University of Helsinki and Helsinki University Hospital, Research Program in Systems Oncology, Faculty of Medicine, University of Helsinki, Helsinki, Finland; 11 Department of Radiology, Brigham and Women’s Hospital, Dana-Farber Cancer Institute, Harvard Medical School, Boston, Massachusetts

## Abstract

**Importance:**

Sarcopenia is an established prognostic factor in patients with head and neck squamous cell carcinoma (HNSCC); the quantification of sarcopenia assessed by imaging is typically achieved through the skeletal muscle index (SMI), which can be derived from cervical skeletal muscle segmentation and cross-sectional area. However, manual muscle segmentation is labor intensive, prone to interobserver variability, and impractical for large-scale clinical use.

**Objective:**

To develop and externally validate a fully automated image-based deep learning platform for cervical vertebral muscle segmentation and SMI calculation and evaluate associations with survival and treatment toxicity outcomes.

**Design, Setting, and Participants:**

For this prognostic study, a model development data set was curated from publicly available and deidentified data from patients with HNSCC treated at MD Anderson Cancer Center between January 1, 2003, and December 31, 2013. A total of 899 patients undergoing primary radiation for HNSCC with abdominal computed tomography scans and complete clinical information were selected. An external validation data set was retrospectively collected from patients undergoing primary radiation therapy between January 1, 1996, and December 31, 2013, at Brigham and Women’s Hospital. The data analysis was performed between May 1, 2022, and March 31, 2023.

**Exposure:**

C3 vertebral skeletal muscle segmentation during radiation therapy for HNSCC.

**Main Outcomes and Measures:**

Overall survival and treatment toxicity outcomes of HNSCC.

**Results:**

The total patient cohort comprised 899 patients with HNSCC (median [range] age, 58 [24-90] years; 140 female [15.6%] and 755 male [84.0%]). Dice similarity coefficients for the validation set (n = 96) and internal test set (n = 48) were 0.90 (95% CI, 0.90-0.91) and 0.90 (95% CI, 0.89-0.91), respectively, with a mean 96.2% acceptable rate between 2 reviewers on external clinical testing (n = 377). Estimated cross-sectional area and SMI values were associated with manually annotated values (Pearson *r* = 0.99; *P* < .001) across data sets. On multivariable Cox proportional hazards regression, SMI-derived sarcopenia was associated with worse overall survival (hazard ratio, 2.05; 95% CI, 1.04-4.04; *P* = .04) and longer feeding tube duration (median [range], 162 [6-1477] vs 134 [15-1255] days; hazard ratio, 0.66; 95% CI, 0.48-0.89; *P* = .006) than no sarcopenia.

**Conclusions and Relevance:**

This prognostic study’s findings show external validation of a fully automated deep learning pipeline to accurately measure sarcopenia in HNSCC and an association with important disease outcomes. The pipeline could enable the integration of sarcopenia assessment into clinical decision making for individuals with HNSCC.

## Key Points

**Question:**

Can a deep learning model that uses standard-of-care head and neck computed tomography (CT) data assess sarcopenia in patients with head and neck squamous cell carcinoma (HNSCC) and predict overall survival and disease outcome?

**Findings:**

In this prognostic study of CT data from 899 patients with HNSCC, a deep learning pipeline accurately segmented the C3 skeletal muscle area to derive a skeletal muscle index for sarcopenia. The sarcopenia status was associated with a significantly reduced overall survival and increased risk of feeding tube dependency.

**Meaning:**

These findings represent the first externally and clinician-validated automated sarcopenia assessment deep learning pipeline for HNSCC, which may inform treatment decisions and triage of patients.

## Teaser

This prognostic study validates a deep learning platform for sarcopenia analysis in head and neck cancer and evaluates survival and treatment toxicity outcomes.

## Introduction

Head and neck squamous cell carcinoma (HNSCC) is the sixth most common cancer worldwide.^1^ Primary treatment approaches include surgery, radiation therapy, and chemotherapy, with a multimodality approach generally needed for advanced disease.^2^ Although HNSCC can be cured, treatment often results in substantial acute and long-term toxic outcomes.^3^ Sarcopenia, a skeletal muscle disorder characterized by age-associated decreased muscle function and reduced skeletal muscle mass, results from several factors, including aging, malnutrition, inactivity, neurologic disorders, and cancer.^4,5^ Progressive sarcopenia is a component of cancer cachexia, a multifactorial syndrome that leads to functional decline that is difficult to reverse and leads to early mortality.^6^ Both sarcopenia and cachexia are negative prognostic indicators in many forms of cancer.^7^ Patients with HNSCC are particularly susceptible to sarcopenia due to disease- and treatment-related malnutrition and dysphagia.^8,9^

Computed tomography (CT) is a well-established method for quantifying body composition and has been used extensively in clinical research.^10^ Imaging-assessed sarcopenia typically has been performed by measurement of skeletal muscle at the L3 vertebra.^4,11,12^ However, routine CT imaging for HNSCC does not extend to the abdomen, substantially limiting the feasibility of monitoring sarcopenia in these patients. To overcome this limitation, a series of studies have proposed a new method of estimating sarcopenia that uses skeletal muscle at the C3 vertebral level and have shown a strong correlation with validated standard L3 skeletal muscle assessment.^9,13,14^

Currently, calculation of the skeletal muscle index (SMI) through C3 muscle segmentation relies primarily on manual or semiautomated techniques,^9,13,14,15,16,17^ which can be time consuming^18^ and is prone to error and intra- and interobserver variability.^19,20^ Accurate manual segmentation requires input from clinical experts with specialized knowledge of the head and neck, as well as specific training for reproducible C3 muscle segmentation and access to segmentation software. A fully automated, accurate SMI assessment pipeline is thus necessary for clinical integration and utility in the management and monitoring of HNSCC. In past years, multiple deep learning models have been created and extensively used for medical imaging.^21,22,23,24,25,26^ Although recent studies have applied deep learning techniques to determine skeletal muscle through abdominal CT scans on the L3 vertebral level,^27,28,29^ few have been performed in head and neck cancer, a disease that has been increasing in prevalence and is known for its challenges in terms of patient vulnerability, treatment decisions, and long-term adverse effects. Recently, Naser et al^30^ introduced a multistage deep learning approach for segmenting the C3 vertebral region using head and neck CT scans, which showed good model performance and a potential for predicting patient survival. However, the study was confined to a single institution and lacked external validation and clinical evaluation, thus limiting clinical translatability.^30^

In this study, we hypothesized that a fully automated, multistage deep learning pipeline could be developed and externally validated for skeletal muscle segmentation to calculate SMI and assess sarcopenia. We also evaluated the clinical relevance of these measurements by assessing the prognostic value of baseline quantification of sarcopenia and its association with survival and toxicity in patients undergoing treatment. By automating the process of imaging-assessed sarcopenia, we sought to generate fast, consistent, and precise measurements to facilitate clinical translation and guide clinical decision making for patients with HNSCC.

## Methods

### Study Cohort

This prognostic study was approved by the Mass General Brigham institutional review board with a waiver of informed consent given the retrospective nature of the research and that many included patients were since deceased or no longer followed at the study institution. The study was conducted in accordance with the Declaration of Helsinki and follows the Transparent Reporting of a Multivariable Prediction Model for Individual Prognosis or Diagnosis (TRIPOD) reporting guideline.^31^

**Figure 1.**
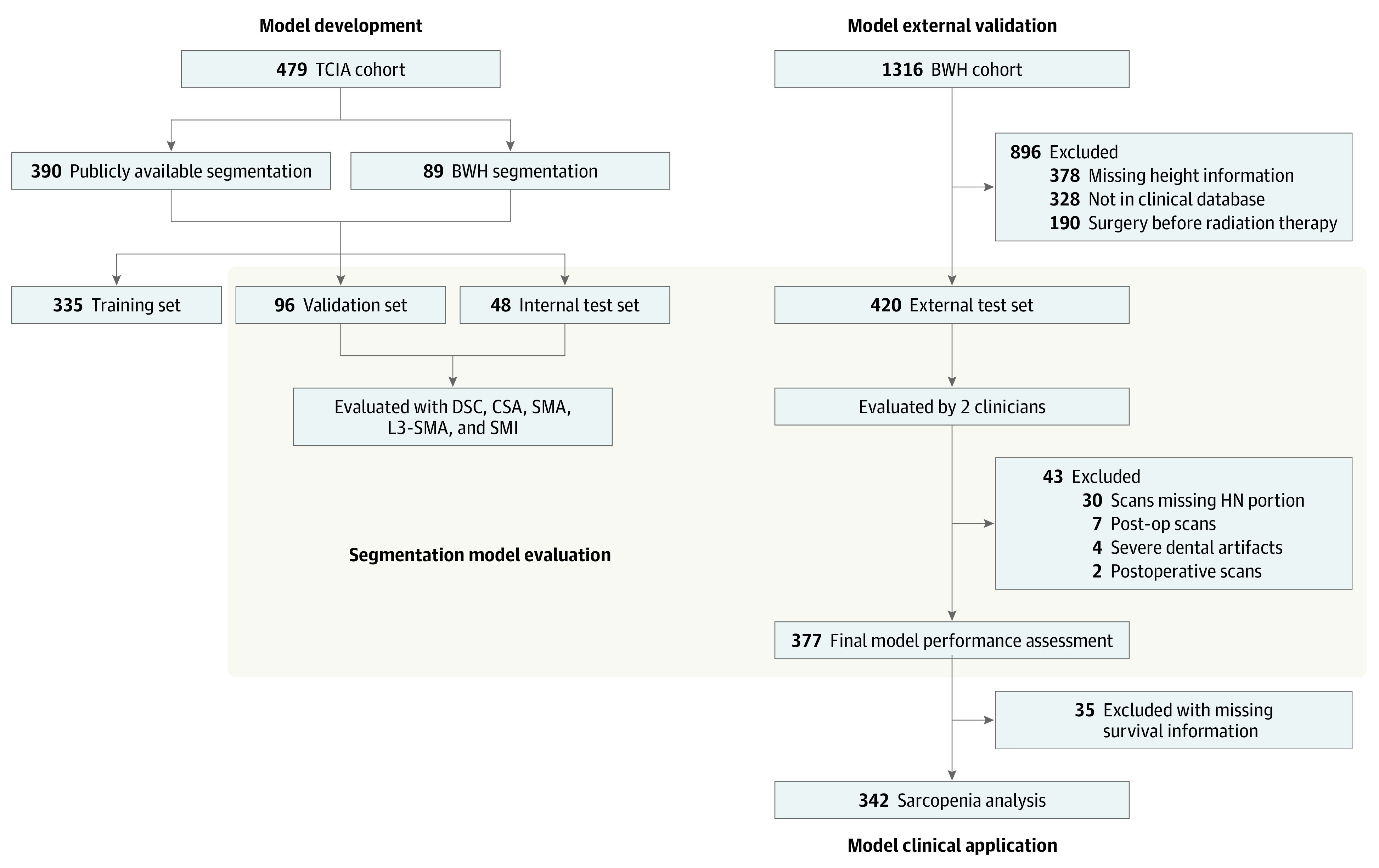
Flow Diagrams for Training, Validation, and Internal Test and External Test Data Sets BWH indicates Brigham and Women’s Hospital; CSA, cross-sectional area; DSC, Dice similarity coefficient; HN, head and neck; SMA, skeletal muscle area; SMI, skeletal muscle index; TCIA, The Cancer Imaging Archive.

The model development data set (n = 479) was curated via The Cancer Imaging Archive^32^ from publicly available, deidentified data from patients treated at the MD Anderson Cancer Center (MDACC) between January 1, 2003, and December 13, 2013. Ground truth segmentations for the development data set (n = 390) were obtained, in part, from a publicly available data set (n = 301).^33^ The remainder of the images in the development data set (n = 89) were manually segmented by an experienced radiation oncologist (A.S.). For external validation, 1316 consecutive patients who underwent primary radiation therapy for HNSCC between January 1, 1996, and December 31, 2013, were retrospectively collected from Brigham and Women’s Hospital (BWH). A total of 988 patients who had complete abdominal CT scans and linked clinical data were included. Subsequently, additional patients were excluded due to missing clinical database information (n = 328), surgery before radiation therapy (n = 190), and missing height information (n = 378), leading to a final set of 420 patients used in the external set (Figure 1). All images were standard diagnostic CT scans (eTables 1 and 2 in Supplement 1). A series of curation and preprocessing procedures were performed to ensure that all data met the quality and standard requirement of the segmentation and predictive models (Figure 1).

### Image Segmentation and Preprocessing

C3 skeletal muscle was manually delineated at the middle of the C3 vertebral body level and contoured in 1 slice to include the paraspinal muscles, scalene muscles, and sternocleidomastoid muscle. No pathologic tissue was included in the skeletal muscle volume. Image preprocessing was conducted using Python, version 3.8 software (Python Software Foundation) and underwent image format conversion, normalization, interpolation, and cropping. Detailed image preprocessing steps are found in the eMethods in Supplement 1.

### Model Development, Training, and Validation

To build an efficient, fully automated pipeline for accurate C3 segmentation, we adopted a 2-stage deep learning approach: (1) slice selection corresponding to the mid-C3 vertebral body in the axial plane and (2) segmentation of the skeletal muscle (eFigure 1 in Supplement 1). The slice selection step used a DenseNet,^34^ and the segmentation step used a U-Net.^35^ The development data set (n = 479) was randomly split into a training set (n = 335), validation set (n = 96), and test set (n = 48). To reduce model overfitting in training, we used data augmentation strategies, including small random translations and rotations. To assess model stability, we performed a 5-fold cross validation. The models were trained for 100 epochs with an initial learning rate of 0.005. To achieve optimal training and validation performance, model hyperparameters were chosen as recommended in a full-body composition study by Bridge et al.^36^ All models were trained from scratch using TensorFlow, version 2.8 in Python. Detailed model architectures, including development, training, and validation processes, are found in eFigures 2 and 3 in Supplement 1.

### External Validation

To determine whether the model could be generalized to patients from outside institutions, we used the BWH data set (n = 420) for the external test. Two experienced radiation oncologists (F.H. and B.H.K.) individually reviewed and evaluated the quality of model-generated C3 muscle segmentations by using a Likert scale from 0 to 3 to generate a clinical acceptability score, defined as follows: 0, the model selected an incorrect axial slice for segmentation that does not correspond to the C3 vertebral body; 1, the segmentation is unacceptable (defined as an estimated >5% muscle volume discrepancy compared with expert segmentation); 2, the segmentation is clinically acceptable, though when compared with expert segmentation would result in a small volume discrepancy of less than or equal to 5%; and 3, segmentation is acceptable with no difference from expert segmentation.

### Definition of Sarcopenia and Association With Outcomes

L3 skeletal muscle area and SMI were calculated based on the C3 cross-sectional area (CSA), age, sex, weight, and height as proposed by Swartz et al^13^ and van Rijn-Dekker et al^9^ (eMethods in Supplement 1). Race and ethnicity data were not available in the institutional databases used for this analysis. The SMI thresholds of 52.4 cm^2^/m^2^ for males and 38.5 cm^2^/m^2^ for females were adopted to stratify patients into sarcopenia and no sarcopenia groups, as established by Prado et al.^37^ Body mass index (BMI), measured by weight in kilograms divided by height in meters squared, is a commonly used metric to assess the health and prognosis of patients with HNSCC during therapy, though it does not provide an assessment of muscle mass directly. To evaluate whether SMI-based sarcopenia was more predictive than BMI for overall survival (OS) and percutaneous endoscopic gastrostomy (PEG) tube duration, we substituted BMI for sarcopenia in the univariable and multivariable analyses. For primary comparison, we stratified patients into underweight (BMI<18.5) and not underweight (BMI≥18.5) groups based on World Health Organization classification.^38^

### Statistical Analysis

The data analysis was performed between May 1, 2022, and March 31, 2023. The Dice similarity coefficient (DSC), precision, recall, and intraclass correlation coefficient were calculated to assess segmentation model performance. The Kruskal-Wallis rank sum test and Fisher exact test were performed to test for differences among the training, validation, internal test, and external test data sets. The interrater reliability test using the agreement coefficient 1 introduced by Gwet^39^ was used to measure the agreement between the ratings by 2 clinicians (F.H. and B.H.K.) on the acceptability scores. The predictive association of sarcopenia with toxicity end points was evaluated using univariable logistic regression analyses. The sarcopenia associations with OS and PEG tube duration were assessed using Cox proportional hazards regression.^38^ We compared model fit using BMI in place of sarcopenia with absolute change in Akaike information criterion (AIC) and bayesian information criterion (BIC). All statistical metrics and curves were generated using scikit-learn, SciPy, and Lifelines packages in Python, version 3.8 and Stata, version 17.0 (StataCorp LLC) software. A 2-sided *P* < .05 was considered statistically significant.

## Results

### Patient Characteristics

The total patient cohort comprised 899 patients with HNSCC (Table 1). The median age of the patients was 58 years (range, 24-90 years). Most of the patients were male (755 [84.0%] vs 140 female [15.6%] and 4 unspecified [0.5%]). The primary cancer site was most commonly the oropharynx (760 [84.5%]). Most of the patients had stage IV cancer (664 [73.9%]). Human papillomavirus (HPV) p16 status was positive for 434 patients (48.3%), negative for 86 (9.6%), and unspecified for 379 (42.2%).

**Table 1.** Patient Characteristics (N = 899)

	No. (%)				*P* value
	Training (MDACC, n = 335)	Validation (MDACC, n = 96)	Internal test (MDACC, n = 48)	External test (BWH, n = 420)	
Age, median (range), y	57 (24-83)	58 (29-81)	59.5 (41-87)	59 (24-87)	.37^a^
Sex					
Female	41 (12.2)	16 (16.7)	8 (16.7)	75 (17.9)	.32^b^
Male	291 (86.9)	80 (83.3)	40 (83.3)	344 (81.9)	
Unspecified	3 (0.9)	0	0	1 (0.2)	
Smoking status					
Current	68 (20.3)	19 (19.8)	11 (22.9)	55 (13.1)	<.001^b^
Former	95 (28.4)	40 (41.7)	17 (35.4)	217 (51.7)	
Never	103 (30.7)	33 (34.4)	17 (35.4)	145 (34.5)	
Unspecified	67 (20.0)	4 (4.2)	3 (6.2)	3 (0.7)	
Primary cancer site					
Oropharynx	309 (92.2)	92 (95.8)	45 (93.8)	314 (74.8)	<.001^b^
Nasopharynx	1 (0.3)	0	0	0	
Larynx or hypopharynx	2 (0.6)	0	0	76 (18.1)	
Oral cavity	2 (0.6)	0	0	0	
Unknown or other	19 (5.7)	4 (4.2)	3 (6.2)	30 (7.1)	
AJCC stage					
I	3 (0.9)	1 (1.0)	0	12 (2.9)	.001^b^
II	11 (3.3)	3 (3.1)	2 (4.2)	34 (8.1)	
III	42 (12.5)	16 (16.7)	12 (25.0)	75 (17.9)	
IV	266 (79.4)	72 (75.0)	31 (64.6)	295 (70.2)	
Unspecified	13 (3.9)	4 (4.2)	3 (6.2)	4 (1.0)	
HPV p16 status^c^					
Negative	19 (5.7)	9 (9.4)	9 (18.8)	49 (11.7)	<.001^b^
Positive	98 (29.3)	76 (79.2)	36 (75.0)	224 (53.3)	
Unspecified	218 (65.1)	11 (11.5)	3 (6.2)	147 (35.0)	
T stage					
T0	2 (0.6)	0	0	0	.24^b^
T1	56 (16.7)	20 (20.8)	11 (22.9)	89 (21.2)	
T2	143 (42.7)	35 (36.5)	17 (35.4)	160 (38.1)	
T3	77 (23.0)	22 (22.9)	10 (20.8)	109 (26.0)	
T4	44 (13.1)	15 (15.6)	7 (14.6)	57 (13.6)	
Unspecified	13 (3.9)	4 (4.2)	3 (6.2)	5 (1.2)	
N stage					
N0	33 (9.9)	9 (9.4)	5 (10.4)	85 (20.2)	<.001^b^
N1	31 (9.3)	13 (13.5)	9 (18.8)	52 (12.4)	
N2	246 (74.3)	68 (70.8)	31 (64.6)	247 (58.8)	
N3	12 (3.6)	2 (2.1)	0	31 (7.4)	
Unspecified	13 (3.9)	4 (4.2)	3 (6.2)	5 (1.2)	

Abbreviations: AJCC, American Joint Committee on Cancer (7th ed); BWH, Brigham and Women’s Hospital; HPV, human papillomavirus; MDACC, MD Anderson Cancer Center.

^a^ Kruskal-Wallis rank sum test.

^b^ Fisher exact test for differences among the data sets.

^c^ Patients with nonoropharyngeal carcinoma who did not undergo HPV p16 testing were coded as negative, given the very low incidence of HPV p16-positive tumors in these disease sites.

### Model Performance

**Figure 2.**
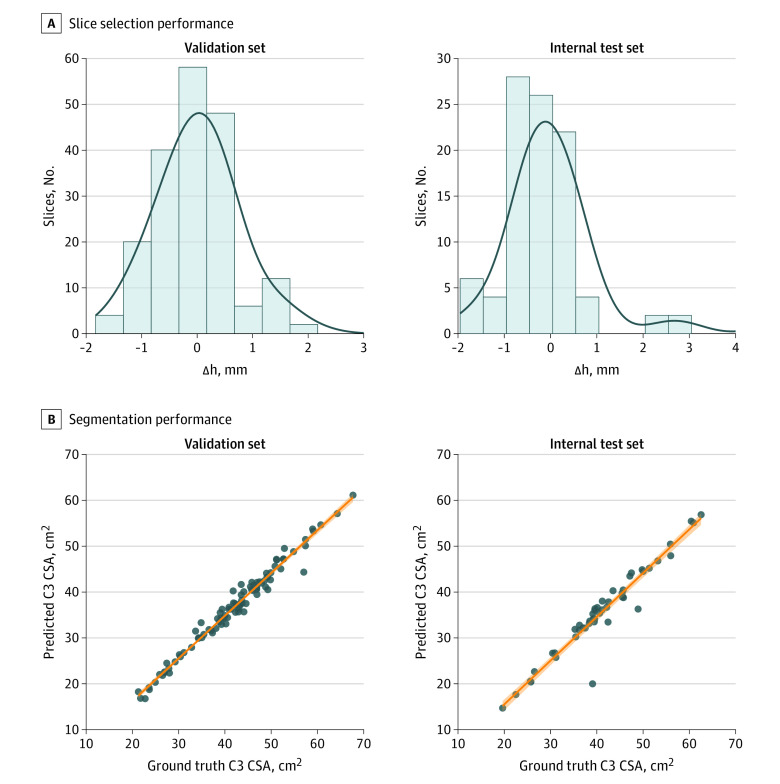
Performance of the Convolutional Neural Network Slice Selection Model and U-Net Segmentation Model for Segmentation of the C3 Vertebral Section A) Histogram shows the difference (Δh) between the location of the model-estimated C3 section slice and the location of the ground truth manually segmented computed tomography slice for the validation set (mean [SD] Δh, 0.11 [1.13] mm) and internal test set (mean [SD] Δh, 0.07 [1.08] mm). B) Scatterplots depict the C3 skeletal muscle cross-sectional area (CSA), with the ground truth manual segmentation on the x-axis and the calculated CSA using estimated segmentations on the y-axis for validation (*r* = 0.99; *P* < .001) and internal test (*r* = 0.96; *P* < .001) sets.

**Figure 3.**
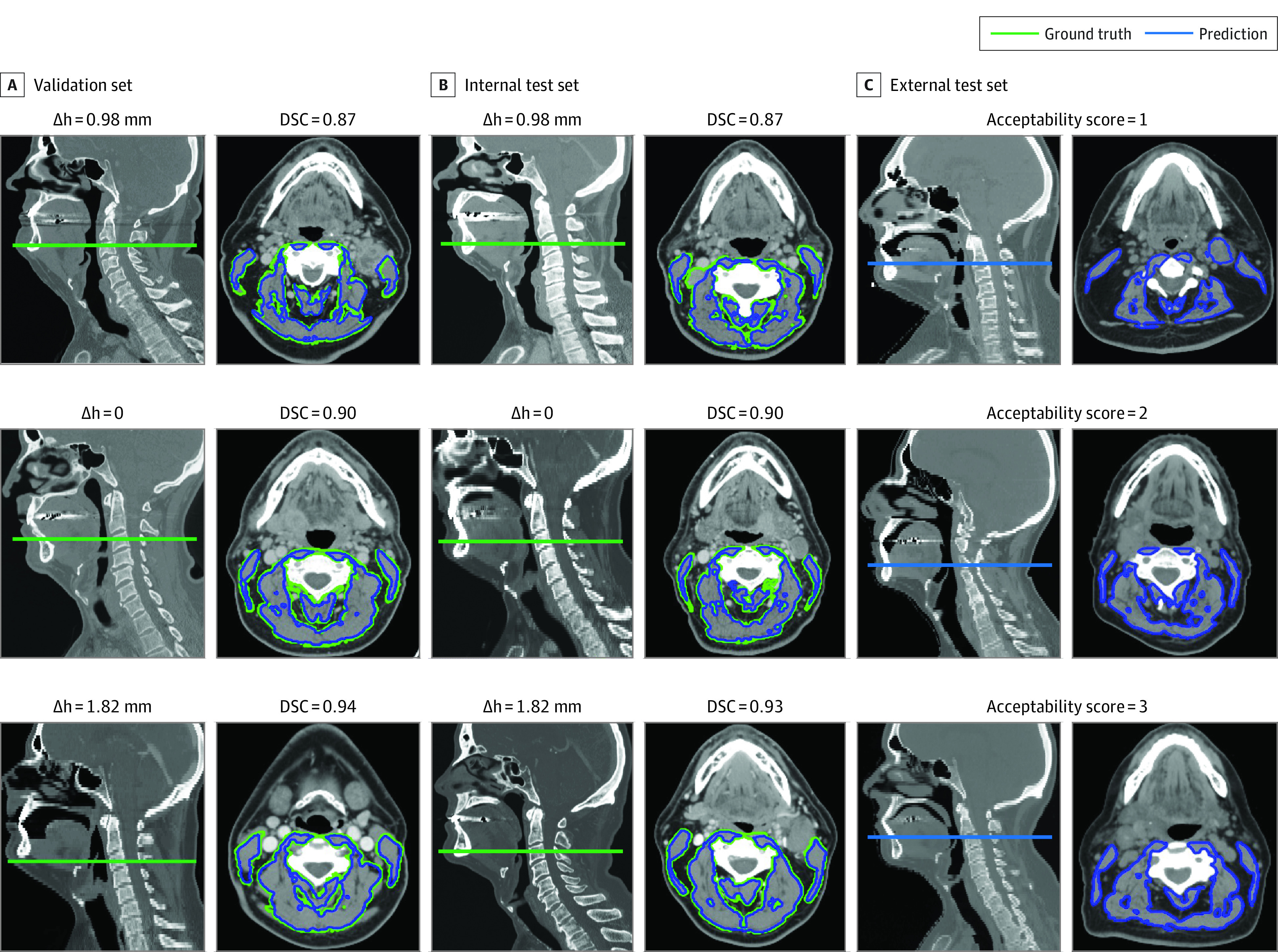
Representative Cases With Ground Truth Slices of C3 Vertebral Sections on Sagittal Computed Tomography (CT) Images and Ground Truth Segmentations on Axial Images Estimated segmentations with varying Dice similarity coefficient (DSC) values (greater than, equal to, or less than the median DSC) were overlaid on axial CT images to show their similarities to ground truth segmentations for validation and internal test sets. Corresponding difference (Δh) between the estimated C3 section slice and ground truth slice and DSC values are annotated for each case in the validation and internal test sets. Model-estimated C3 slices on sagittal CT images and segmentation on axial CT images are also shown for the external test set. Acceptability scores from expert clinicians’ review are annotated for corresponding cases in the external test set.

Evaluation of model slice selection revealed that the difference between the estimated mid-C3 slice and the ground truth slice was minimal, as shown by histogram analysis (Figure 2A). The mean (SD) difference between the locations of predicted C3 slice and ground truth slice were 0.11 (1.13) mm and 0.07 (1.08) mm for the validation and internal test sets, respectively (Figure 2A). The DSC values obtained for the validation and internal test sets predicted segmentations vs ground truth were 0.90 (95% CI, 0.90-0.91) and 0.90 (95% CI, 0.89-0.91), respectively (eFigure 5A and eTable 4 in Supplement 1). Additionally, the precision, recall, and intraclass correlation coefficient scores, as summarized in Figure 2B, all showed excellent model performance in predicting C3 segmentations. The C3 CSAs derived from predicted segmentations showed near-perfect correlations with the ground truth–calculated CSAs (validation set: *r* = 0.99 [*P* < .001]; test set: *r* = 0.96 [*P* < .001]) (Figure 2B). Representative examples of C3 section slices on sagittal CT images and ground truth segmentations on axial images with performance metrics are shown in Figure 3.

### External Test

The reviewers conducted initial quality assessment for the external test set (n = 420) and identified 43 scans (10%) they judged to be problematic, resulting in a final set of 377 patients, which was then carefully reviewed and assigned acceptability scores. Representative cases with C3 slice predictions and ground truth segmentations for acceptability scores 0, 1, 2, and 3 are shown in Figure 3C. The review scores are summarized in eFigure 5B in Supplement 1, with 183 cases (48.5%) and 188 cases (49.9%) deemed acceptable with minor changes or no changes needed, respectively, for reviewer 1 and with 199 cases (52.8%) and 161 cases (42.7%) deemed acceptable with minor changes or no changes needed, respectively, for reviewer 2 (mean acceptable rate, 96.2%). The interrater agreement between the 2 reviewers was excellent, with an agreement coefficient 1 score of 0.94. The segmentation acceptability rate was comparable across patient sex, age, and smoking status for both reviewers (eTable 15 in Supplement 1). We further investigated the unacceptable segmentations that were given by either one of the reviewers. We identified 23 cases (6.1%), with 11 (2.9%) from reviewer 1 and 18 (4.8%) from reviewer 2. Given high overall acceptability, we moved forward with the SMI calculation and designation of sarcopenia for the external test set. Detailed failure analyses are found in the eMethods, eTable 3, and eFigure 4 in Supplement 1.

### Skeletal Muscle Index Measurement Comparisons

We calculated and compared the SMI values between model predictions and ground truth for both the validation set and internal test set (eFigure 6A and B in Supplement 1). Accurate model skeletal muscle segmentations led to predicted SMI values that were highly correlated with the ground truth values (Pearson *r* ≥ 0.99; *P* < .001) for both female and male patients in all data sets (eFigure 6A and B in Supplement 1).

### Predictive Analyses for Sarcopenia

A total of 342 patients with complete survival and toxicity information from the external test set were further included for sarcopenia predictive analysis (eTable 5 in Supplement 1). The median follow-up was 43 months (range, 1-170 months), and the OS rate at 5 years was 80.7%. The 5-year survival rate was 84.4% in patients without sarcopenia vs 73.1% in patients with sarcopenia (hazard ratio [HR], 2.21; 95% CI, 1.08-4.12; *P* = .03) (eFigure 6C in Supplement 1). A total of 58 patients with sarcopenia and 9 without sarcopenia were dead at the follow-up time of 72 months. On multivariable analysis, variables associated with worse OS were sarcopenia (HR, 2.05; 95% CI, 1.04-4.04; *P* = .04), Adult Comorbidity Evaluation 27 score of 2 or higher (HR, 2.03; 95% CI, 1.24-3.23; *P* = .005), nonoropharynx tumor site (HR, 3.66; 95% CI, 2.20-6.09; *P* < .001), and T3 to T4 stage (HR, 2.29; 95% CI, 1.42-3.68]; *P* = .001), but not age 65 years or older, smoking history of 10 pack-years or more, N2 to N3 stage, or stage III to IV disease (Table 2). Sarcopenia vs no sarcopenia was associated with longer PEG tube duration (median, 162 [range, 6-1477] vs 134 [range, 15-1255] days, respectively; HR, 1.67; 95% CI, 1.23-2.22; *P* = .001) (Table 2; eFigure 6D in Supplement 1). On multivariable analysis, variables associated with longer PEG tube duration were sarcopenia (HR, 0.66; 95% CI, 0.48-0.89; *P* = .006) and Adult Comorbidity Evaluation 27 score of 2 or higher (HR, 0.72; 95% CI, 0.53-0.97; *P* = .03) (Table 2). Sarcopenia was not associated with insertion of a PEG tube at diagnosis but was associated with a higher risk of having a PEG tube at last follow-up (odds ratio, 2.25; 95% CI, 1.02-4.99; *P* = .046) (eTable 6 in Supplement 1). Sarcopenia was not associated with a higher risk of hospitalization less than 3 months after radiation therapy, a higher risk of osteoradionecrosis, post–radiation therapy stricture, or treatment complication requiring surgery. On subgroup analysis of only patients with known HPV status (n = 225), sarcopenia was associated with survival and PEG tube duration on univariable analysis but not on multivariable analysis (eTables 13 and 14 in Supplement 1).

**Table 2.** Univariable and Multivariable Analyses for Overall Survival and Percutaneous Endoscopic Gastrostomy (PEG) Tube Duration

	Overall survival				PEG tube duration^a^			
	Univariable		Multivariable		Univariable		Multivariable	
	HR (95% CI)	*P* value	HR (95% CI)	*P* value	HR (95% CI)	*P* value	HR (95% CI)	*P* value
Sarcopenia								
No	1 [Reference]	NA	1 [Reference]	NA	1 [Reference]	NA	NA	NA
Yes	2.11 (1.08-4.12)	.03	2.05 (1.04-4.04)	.04	0.60 (0.45-0.80)	.001	0.66 (0.48-0.89)	.006
Age, y								
<65	1 [Reference]	NA	1 [Reference]	NA	1 [Reference]	NA	NA	NA
≥65	1.93 (1.20-3.10)	.007	1.07 (0.65-1.77)	.79	0.68 (0.51-0.91)	.009	0.80 (0.58-1.09)	.15
Smoking history, pack-y								
<10	1 [Reference]	NA	1 [Reference]	NA	1 [Reference]	NA	NA	NA
≥10	2.00 (1.23-3.25)	.005	1.09 (0.64-1.84)	.75	0.60 (0.47-0.77)	<.001	0.67 (0.51-0.87)	.002
ACE-27 score								
0-1	1 [Reference]	NA	1 [Reference]	NA	1 [Reference]	NA	NA	NA
2-3	2.24 (1.39-3.62)	.001	2.03 (1.24-3.23)	.005	0.70 (0.52-0.95)	.02	0.72 (0.53-0.97)	.03
Tumor site								
Oropharynx primary	1 [Reference]	NA	1 [Reference]	NA	1 [Reference]	NA	NA	NA
Nonoropharynx	3.92 (2.45-6.25)	<.001	3.66 (2.20-6.09)	<.001	0.64 (0.45-0.90)	.01	0.80 (0.56-1.14)	.22
T stage								
T1-T2	1 [Reference]	NA	1 [Reference]	NA	1 [Reference]	NA	NA	NA
T3-T4	2.36 (1.47-3.77)	<.001	2.29 (1.42-3.68)	.001	0.71 (0.56-0.92)	.008	0.78 (0.60-1.01)	.06
N stage								
N0-N1	1 [Reference]	NA	NA	NA	1 [Reference]	NA	NA	NA
N2-N3	0.88 (0.54-1.43)	.60	NA	NA	1.12 (0.84-1.48)	.43	NA	NA
AJCC stage								
I-II	1 [Reference]	NA	NA	NA	1 [Reference]	NA	NA	NA
III-IV	1.31 (0.69-2.49)	.41	NA	NA	0.61 (0.34-1.12)	.11	NA	NA

Abbreviations: ACE-27, Adult Comorbidity Evaluation 27; AJCC, American Joint Committee on Cancer (7th ed); HR, hazard ratio; NA, not applicable.

^a^ PEG tube duration was defined as the time from insertion to removal of PEG tube (ie, HR<1.00 represents longer time to removal or greater PEG tube duration).

### Sarcopenia and BMI

The SMI showed a significant positive correlation (Pearson *r* = 0.67; *P* < .001) with BMI for all patients (n = 342). Overall survival was associated with underweight (BMI<18.5) in univariable analysis (HR, 4.70; 95% CI, 1.70-12.90; *P* = .003) (eTable 7 in Supplement 1) but not in multivariable analysis (HR, 2.01; 95% CI, 0.71-5.74; *P* = .19) (eTable 7 in Supplement 1). Furthermore, in the underweight-based model, AIC and BIC values were higher than in the sarcopenia-based model (AIC, 703.6 vs 700.0 [Δ = 3.6]; BIC, 726.5 vs 722.9 [Δ = 4.0]) (eTable 9 in Supplement 1), suggesting that the sarcopenia-based model had an improved fit for OS. Similarly, PEG tube duration was not associated with underweight in multivariable analysis (HR, 0.97; 95% CI, 0.36-2.64; *P* = .95) (eTable 10 in Supplement 1), and the underweight-based model had both higher AIC and BIC values (AIC, 2489.3 vs 2482.3 [Δ = 7.0]; BIC, 2511.4 vs 2504.4 [Δ = 7.0]) (eTable 12 in Supplement 1), suggesting an improved fit of the sarcopenia-based model for PEG tube duration. Sensitivity analysis using an overweight BMI threshold of 25 yielded similar results (eTables 8 and 11 in Supplement 1).

## Discussion

In this study, we successfully developed and validated an end-to-end deep learning pipeline that uses head and neck CT images for efficient and accurate segmenting of cervical vertebral skeletal muscle, calculation of SMI, and diagnosis of imaging-assessed sarcopenia in patients with HNSCC. We applied our tool to a large external validation cohort, where we found that imaging-based sarcopenia was associated with poorer OS and longer PEG tube duration. Furthermore, sarcopenia was more predictive of these outcomes than BMI. This externally validated deep learning pipeline could translate clinically as a fast and fully automated prognostic tool for patients with HNSCC in routine clinical practice. This end-to-end deep learning pipeline is the first, in our knowledge, for determining sarcopenia that uses head and neck CT images and that has been externally validated with a substantial patient population.

We followed a 2-step process, similar to a recent study conducted by Naser et al,^30^ to segment the C3 skeletal muscle. However, our methods differ substantially. Naser et al used a 3-dimensional (3D) ResUNet model to segment the C3 vertebral section first and then automatically selected the middle slice and applied a 2D ResUNet model to segment the skeletal muscle. In contrast, we used a 2D DenseNet-based regression model to automatically select the C3 skeletal muscle slice, and then used a 2D U-Net model to segment the selected slice. We achieved excellent model performance for both the slice selection and segmentation models in the validation and internal test sets. In a large external test set, 96.2% of skeletal muscle segmentations was also deemed acceptable by expert consensus review. Compared with 3D convolutional neural network models, 2D convolutional neural network models are generally much easier to train and implement, making our pipeline fast and efficient for the C3 skeletal muscle segmentation for sarcopenia analysis. In this study, it took our experienced radiation oncologist 5 to 10 minutes to identify and segment C3 skeletal muscle for 1 patient. In contrast, our end-to-end deep learning pipeline required only 0.15 seconds for the same task, which is considerably quicker than a human expert.

Sarcopenia is an important prognostic factor of decreased OS in various types of cancers.^4,5,37^ For HNSCC, these findings appear to be irrespective of geographic area, head and neck tumor sites, and treatment approaches.^40,41^ We found sarcopenia to be associated with worse OS, similar to prior studies.^9,14,15^ In addition to the poorer survival of patients with sarcopenia, there is an increased risk of toxicity after treatment.^9,15,41,42,43^ Radiation therapy to the head and neck region is widely known to induce severe toxic outcomes, such as mucositis, odynophagia, and xerostomia, leading to critical weight loss and malnutrition.^3,15,16,17^ Although chemotherapy is not a primary treatment for HNSCC, it is often given with radiation therapy in either an adjuvant or a definitive setting. Chemotherapy may also be administered prior to other treatments as a neoadjuvant approach. Recent retrospective studies in patients with locally advanced HNSCC concluded that pretreatment of sarcopenia was an important factor associated with chemotherapy dose-limiting toxicity in patients treated with chemoradiation therapy using platinum-based chemotherapy.^44,45^ In our study, we tested the correlations between sarcopenia and a series of chemotherapy and radiation therapy toxicity end points. We found that sarcopenia was associated with longer PEG tube duration and a higher risk of having a PEG tube at last follow-up. This finding validates a study by Karsten et al^43^ that showed that sarcopenia contributes to the risk of prolonged feeding tube dependency in patients with HNSCC treated with primary chemoradiation therapy. We did not find that sarcopenia was associated with a higher risk of hospitalization less than 3 months after radiation therapy. We did not see an association between sarcopenia with risk of osteoradionecrosis and post–radiation therapy stricture. In HNSCC surgical populations, sarcopenia has been shown to be a negative prognostic indicator for both overall complications and wound complications, including pharyngocutaneous fistulas in patients undergoing total laryngectomy for HNSCC.^12^ In our study, however, sarcopenia was not associated with chemoradiation-associated treatment complications requiring surgery.

### Limitations

Our study had several limitations. First, the analysis is limited by the inherent constraints of a retrospective study. Due to various exclusion criteria, a number of patients were excluded from the final analysis, which may bias the distribution of patient characteristics. Our study consisted of patients whose cancers were managed nonoperatively; thus, validity of C3-based sarcopenia in surgically managed cancers requires further study. Our cohort was highly enriched for oropharynx carcinoma, and while we have no reason to believe that mucosal subsite would affect the accuracy of C3 skeletal muscle segmentation, it may modify the effect of sarcopenia as a prognostic factor. Second, our median DSC scores were lower than those reported by Naser et al^30^ (0.90 vs 0.95). We believe that our lower DSC scores are due to the preprocessing step we implemented to account for significant differences in CT imaging parameters between our development cohort (MDACC) and external test cohort (BWH). We were able to achieve a median DSC of 0.94 for validation and internal test sets in the MDACC cohort without this preprocessing step. However, the robustness of our model was compromised when applied to the external test data set. Moving forward, we plan to further optimize our preprocessing steps to improve the model’s performance while maintaining its generalizability. Third, the overall proportion of patients without sarcopenia, as well as death events, were lower in our external cohort than other studies, and we were unable to find an association of PEG tube duration with other known risk factors of long-term dysphagia, including radiation dose-volume parameters of the pharyngeal constrictor muscles.^46^ Fourth, HPV status information was not available for a substantial proportion of the patients in our external test data set. In a relatively smaller patient cohort with available HPV status, we did not observe significant associations of OS and PEG tube duration with sarcopenia as well as with most other clinical variables (eTables 13 and 14 in Supplement 1). Given that HPV status is a crucial clinical risk factor for patients with HNSCC, it is imperative to include it in the sarcopenia-based outcome prediction model. Therefore, our next step will be to focus on curating additional patient data with HPV status to further validate and refine our model.

## Conclusions

In this prognostic study, we developed and externally validated a fully automated deep learning platform for fast and accurate sarcopenia assessment that can be used on routine head and neck CT imaging. Our model has shown excellent C3 skeletal muscle segmentation capability on data sets from different institutions, with high agreement with an expert clinician’s segmentation and high acceptability rates from expert clinicians’ reviews. Furthermore, our findings show that the model’s estimated SMI strongly correlates with the ground truth SMI and that the SMIs estimated worse OS and longer PEG tube duration in a large HNSCC cohort. If further validated, our end-to-end deep learning pipeline could be incorporated into standard clinical practice for directing future treatment approaches and clinical decision making, as well as for individualized supportive measures, including nutrition guidance and physical therapy.

## Notes

Supplement 1.
eMethods.

eReferences
**eTable 1.** CT Scanner Manufacturers and Models Used for Head and Neck Scans **eTable 2.** Head and Neck CT Scan Characteristic Deviation Table **eTable 3.** Summary on Failing Cases on External Test Set **eTable 4.** U-Net Segmentation Model Performance **eTable 5.** Patient Characteristics for Nonsarcopenic and Sarcopenic Groups **eTable 6.** Univariable Analysis for the Association of Sarcopenia With Various Toxicity End Points **eTable 7.** Baseline Univariable and Multivariable Analyses for Overall Survival With Underweight **eTable 8.** Baseline Univariable and Multivariable Analyses for Overall Survival With Overweight **eTable 9.** AIC and BIC for Overall Survival Model **eTable 10.** Multivariable Analyses for PEG Tube Duration With Overweight **eTable 11.** Univariable and Multivariable Analyses for PEG Tube Duration With Overweight **eTable 12.** AIC and BIC for PEG Tube Duration Model **eTable 13.** Univariable and Multivariable Analyses for Overall Survival With Available HPV Status **eTable 14.** Univariable and Multivariable Analyses for PEG Tube Duration With Available HPV Status **eTable 15.** Fairness Assessment of Deep Learning Pipeline **eFigure 1.** Workflow of the Fully Automated Deep Learning Pipeline for Accurate C3 Segmentation **eFigure 2.** Model Architecture for 2D DenseNet-Based Slice Selection Model, Adapted DenseNet CNN Architecture **eFigure 3.** Model Architecture for 2D U-Net–Based Segmentation Model **eFigure 4.** Representative Failing Cases With Different Failing Causes **eFigure 5.** C3 Segmentation Showed Excellent Performance in Both DSC Evaluation and Clinical Acceptability Evaluation **eFigure 6.** Scatter Plots of the Skeletal Muscle Index (SMI) Values and Kaplan-Meier Survival Estimates

Supplement 2.
Data Sharing Statement
