# Supplementary material for: Development and Validation of an Automated Image-Based Deep Learning Platform for Sarcopenia Assessment in Head and Neck Cancer
Source: JAMA Netw Open. 2023 Aug 10;6(8):e2328280. doi: 10.1001/jamanetworkopen.2023.28280 (PMC10415962; doi:10.1001/jamanetworkopen.2023.28280)
Supplement: Supplement 1. — eMethods. eReferences eTable 1. CT Scanner Manufacturers and Models Used for Head and Neck Scans eTable 2. Head and Neck CT Scan Characteristic Deviation Table eTable 3. Summary on Failing Cases on External Test Set eTable 4. U-Net Segmentation Model Performance eTable 5. Patient Characteristics for Nonsarcopenic and Sarcopenic Groups eTable 6. Univariable Analysis for the Association of Sarcopenia With Various Toxicity End Points eTable 7. Baseline Univariable and Multivariable Analyses for Overall Survival With Underweight eTable 8. Baseline Univariable and Multivariable Analyses for Overall Survival With Overweight eTable 9. AIC and BIC for Overall Survival Model eTable 10. Multivariable Analyses for PEG Tube Duration With Overweight eTable 11. Univariable and Multivariable Analyses for PEG Tube Duration With Overweight eTable 12. AIC and BIC for PEG Tube Duration Model eTable 13. Univariable and Multivariable Analyses for Overall Survival With Available HPV Status eTable 14. Univariable and Multivariable Analyses for PEG Tube Duration With Available HPV Status eTable 15. Fairness Assessment of Deep Learning Pipeline eFigure 1. Workflow of the Fully Automated Deep Learning Pipeline for Accurate C3 Segmentation eFigure 2. Model Architecture for 2D DenseNet-Based Slice Selection Model, Adapted DenseNet CNN Architecture eFigure 3. Model Architecture for 2D U-Net–Based Segmentation Model eFigure 4. Representative Failing Cases With Different Failing Causes eFigure 5. C3 Segmentation Showed Excellent Performance in Both DSC Evaluation and Clinical Acceptability Evaluation eFigure 6. Scatter Plots of the Skeletal Muscle Index (SMI) Values and Kaplan-Meier Survival Estimates [file jamanetwopen-e2328280-s001.pdf]

Supplemental Online Content

Ye Z, Saraf A, Ravipati Y, et al. Development and validation of an automated image-based deep learning platform for sarcopenia assessment in head and neck cancer. *JAMA Netw Open*. 2023;6(8):e2328280. doi:10.1001/jamanetworkopen.2023.28280

eMethods

eReferences

eTable 1. CT Scanner Manufacturers and Models Used for Head and Neck Scans

eTable 2. Head and Neck CT Scan Characteristic Deviation Table

eTable 3. Summary on Failing Cases on External Test Set

eTable 4. U-Net Segmentation Model Performance

eTable 5. Patient Characteristics for Nonsarcopenic and Sarcopenic Groups

eTable 6. Univariable Analysis for the Association of sarcopenia With Various Toxicity End Points

eTable 7. Baseline Univariable and Multivariable Analyses for Overall Survival With Underweight

eTable 8. Baseline Univariable and Multivariable Analyses for Overall Survival With Overweight

eTable 9. AIC and BIC for Overall Survival Model

eTable 10. Multivariable Analyses for PEG Tube Duration With Overweight

eTable 11. Univariable and Multivariable Analyses for PEG Tube Duration With Overweight

eTable 12. AIC and BIC for PEG Tube Duration Model

eTable 13. Univariable and Multivariable Analyses for Overall Survival With Available HPV Status

eTable 14. Univariable and Multivariable Analyses for PEG Tube Duration With Available HPV Status

eTable 15. Fairness Assessment of Deep Learning Pipeline

eFigure 1. Workflow of the Fully Automated Deep Learning Pipeline for Accurate C3 Segmentation

eFigure 2. Model Architecture for 2D DenseNet-Based Slice Selection Model, Adapted DenseNet CNN Architecture

eFigure 3. Model Architecture for 2D U-Net–Based Segmentation Model

eFigure 4. Representative Failing Cases With Different Failing Causes

eFigure 5. C3 Segmentation Showed Excellent Performance in Both DSC Evaluation and Clinical Acceptability Evaluation

eFigure 6. Scatter Plots of the Skeletal Muscle Index (SMI) Values and Kaplan-Meier Survival Estimates

This supplemental material has been provided by the authors to give readers additional information about their work.

© 2023 Ye Z et al. *JAMA Network Open.*

eMethods

CT scan characteristics

The CT scans were performed on various CT scanner models from two institutions, including GE LightSpeed RT, GE Discovery RT and Siemens Somatom Confidence CT. Scans were diagnostic quality, using 120-140 kVp energy, slice thickness of 1.25-5 mm, and pixel spacing of 0.3-2.4 mm (eTable 1&2).

Curation and preprocessing for CT images and segmentations

Part of the ground truth (GT) segmentations for the development dataset (n=301) were obtained from a publicly available dataset, as outlined in Wahid et al., *Scientific Data* 2022[1]. The GT segmentations were downloaded and converted to Nearly Raw Raster Data (NRRD) format following the instruction provided at: https://github.com/kwahid/C3_sarcopenia_data_descriptor. CT images for the development dataset were downloaded from The Cancer Imaging Archive (TCIA) and were converted from DICOM format to NRRD format via rasterization packages utilizing SimpleITK and plastimatch (https://plastimatch.org) in Python v3.8. For slice selection model and segmentation model, we adopted two different preprocessing strategies. In the slice selection step, CT intensities were first truncated in the range of [−175, 275] Hounsfield units to increase soft tissue contrast and then normalized to the range of [-1, 1] scale. Then the 3-dimensional (3D) images were converted to 2-dimensional (2D) Numpy files with corresponding slice indices as model inputs. In the segmentation step, predicted image slice from slice selection step was extracted from normalized CT images. A standard cropping step was then employed on x-y planes. Scans were then resized to 512x512 using linear interpolation via SimpleITK and served as the inputs for the segmentation model. All the preprocessing codes are available at: https://github.com/AIM-KannLab/DeepSarcopenia.

Deep learning model development and implementation

To build an efficient fully-automated pipeline for accurate C3 segmentation, we adopted a two-stage DL approach, consisting of a slice selection step and a segmentation step. Specifically, the slice selection step predicts the C3 image slice from the input 3D CT scan and the segmentation step generates the SM segmentation on the predicted C3 slice. The DenseNet architecture (eFigure 2), known for its impressive classification performance[2], was utilized for training the slice selection model. Similarly, the U-Net architecture (eFigure 3), widely recognized for its effectiveness in biomedical image segmentation tasks[3], was employed for train the semantic segmentation of C3 SM on the chosen C3 image slice. An overview of the architecture for the fully automated segmentation pipeline is provided in Fig. 2. In the slice selection step, the DenseNet regression model performed slice-wise regression predictions on each axial slice of the 3D CT scan independently, followed by post-processing to output the target C3 slice. The model takes input CT slice series and learns to predict a single continuous valued output representing the offset of that slice from the target C3 slice (z-offset). To adapt the model architecture for regression task, the final fully connected layer with softmax activation was replaced with a fully connected layer with a single output unit and a sigmoid activation function to output a number ranging from 0 to 1. The mean absolute error loss between this output and the regression target, C3 slice with 0 z-offset, was then used as the loss function in model training. In the segmentation step, the predicted C3 slice was passed to the U-Net segmentation model to segment the C3 SM for estimating the cross-sectional areas at the C3 level. In the U-Net structure, batch normalization was added to each activation, and the loss function was changed to soft Dice maximization loss to deal with class imbalances between the muscle mass and the background. All the codes for networks and model training are available at: https://github.com/AIM-KannLab/DeepSarcopenia.

Model training and validation

After data preprocessing, the total development dataset (n=479) was randomly split into training set (n=335), validation set (n=96), and test set (n=48) with a split ratio of 70%:20%:10%. To reduce model overfitting in training, we employed data augmentation strategies including small random translations of up to 0.05 times the image size in both the horizontal and vertical directions, and small rotations of up to 5 degrees in either direction drawn from a uniform distribution. The models were trained for 100 epochs with an initial learning rate of 0.005 that was multiplied by a factor of 0.1 every 25 epochs. To achieve optimal training and validation performance, model hyper-parameters including the number of layers in each dense block of DenseNet, up/down sampling modules, and initial features of U-Net were chosen as recommended in a full body composition study that experimented with a similar architecture by Bridge et al.[4]. A batch size of 16 was used for model training and the Adam's optimizer was used to minimize the loss functions during the training of both models. All models were trained from scratch using TensorFlow v2.8 in Python. The performance of the automated pipeline was evaluated by the placement of the C3 selected slice and the Dice Similarity Coefficient (DSC) of the auto segmentation over ground truth on the validation set. The reliability of the auto segmentations for its use in the sarcopenia determination is evaluated by the Intra Class Correlation (ICC) coefficient of cross-sectional area measurement.

Five-fold cross validation

To assess the model stability, we performed a 5-fold cross validation. The development dataset (n=479) was randomly split into training-validation set (n=431) and internal test set (n=48) using 5-fold cross-validation. The training-validation set was further randomly split into training set (n=335) and validation set (n=96).

Definition of sarcopenia
As proposed by Swartz et al.[5] and van Rijn-Dekker et al.[6], the SMA at the L3 lumbar level was calculated based on Equation 1 and then the SMI was calculated from Equation 2.

$$\text{SMA} = 27.30 + (1.36 \times \text{CSA}) - (0.67 \times \text{age}) + (0.64 \times \text{weight}) + (26.44 \times \text{sex}) \qquad [1]$$

Here SMA is the cross-sectional area in cm^2 at the L3 lumbar level. CSA is the cross-sectional area in cm^2 at the C3 cervical level. Age is the patient's age in years. Weight is the patient's weight in kg. Sex is equal to 1 if the patient is female and is equal to 2 if the patient is male.

$$\text{SMI} = \frac{\text{SMA}}{\text{height}^2} \qquad [2]$$

Here SMI is SMI at the L3 lumber level. SMA is the SMA in cm^2 at the L3 lumbar level calculated by previous formula. Height is the patient's height in meters.

Statistical analysis
HPV status is a known prognostic factor for survival and toxicity in patients with HNSCC. This study included patients when HPV status was not routinely performed and therefore HPV status was unknown for a significant proportion of patients. We approximated this biomarker per establishing stratification by smoking history into two categories: patients with ≤10 pack-year (py) smoking history or patients with>10 py smoking history[7].

Dice similarity coefficient
The Dice similarity coefficient (DSC), also known as the Sørensen–Dice index or simply Dice coefficient, is a statistical tool which measures the similarity between two sets of data – it is essentially a metric that quantifies overlap of two objects. This index has become arguably the most broadly used tool in the validation of image segmentation algorithms. The equation for this concept is:

$$2 * |X \cap Y| / (|X| + |Y|)$$

where X and Y are two sets; |X| means the number of elements in set X; ∩ is used to represent the intersection of two sets and means the elements that are common to both sets.

Results

5-fold cross-validation
we conducted a 5-fold cross-validation (CV) on our development dataset. The results closely aligned with our current findings, as evidenced by Dice DSC (mean±SD) of 0.90±0.06, 0.90±0.06, 0.90±0.06, 0.90±0.07, and 0.90±0.07 for the internal test set across the five folds. These outcomes provided strong validation for the stability and consistency of our model.

Initial quality assessment on external test set
The reviewers conducted initial quality assessment for external test set (n=420) and identified 43 cases (10%) with scans that were judged to be problematic, including scans that were retrieved did not include complete HN area (n=30), scans that showed a postoperative status in the neck (n=7), scans with severe dental artifact (n=4), and scans with skinfold artifact (n=2). After exclusion of the faulty scans, we had a final set of 377 patients, which were then carefully reviewed and assigned with the acceptability scores.

Failure analysis for external test set
We investigated the unacceptable segmentations that were given by either one of the reviewers. We identified 23 cases (6.1%), with 11 cases (2.9%) from reviewer 1 and 18 cases (4.8%) from reviewer 2. Failure modes are summarized in eTable 3, and included 9 cases (39.1%) with sternocleidomastoid (SCM) muscle missing (eFigure 4A); 6 cases (26.1%) with lymph node included (eFigure 4B); 3 cases (13.0%) with posterior neck muscles missing (eFigure 4C); 3 cases (13%) with anterior deep muscle missing (eFigure 4D); 1 case (4.4%) with submental muscle included (eFigure 4E); and 1 case (4.4%) with other muscle included (eFigure 4F).

© 2023 Ye Z et al. *JAMA Network Open.*

Clinical information of patients undergoing sarcopenia analysis

A total of 342 patients with complete survival and toxicity information from the external test set were further included for sarcopenia predictive analysis (eTable 4). The median follow-up for all patients was 43 months and the OS at 5 years was 80.7%. There were 261 (76.3%) sarcopenic patients and 81 (23.7%) non-sarcopenic patients in the dataset. Median age was 59 (range 24-87), most were male (83%), smoking history<10 pack-years (py) (51%), Adult Comorbidity Evaluation 27 (ACE-27) score 0 (39%) or 1 (38%), non-oropharynx primary (73%), and American Joint Committee on Cancer (AJCC) 7th edition stage III (16%), IVa (65%), or IVb (8%).

Fairness assessment of deep learning pipeline

The model exhibited comparable performance across different demographic groups, including females and males, patients older than 65 years and those younger than 65 years, as well as non-smokers and current/former smokers. These findings were consistent for both Reviewer 1 and Reviewer 2, indicating that the model's performance was robust and not significantly influenced by gender, age, or smoking status (eTable15)."

The predictive analysis of sarcopenia on toxicity endpoints

Sarcopenia was not associated with insertion of PEG tube at diagnosis (p=0.12) but was associated with higher risk of having PEG tube at last follow-up (odds ratio (OR) 2.25 [95% CI 1.02-4.99], p=0.05) (eTable 6). Sarcopenia was not significantly associated with higher risk of hospitalization < 3 months after RT (eTable 6; OR 2.18 [95% CI 0.82-5.79], p=0.12). Sarcopenia was not significantly associated with risk of osteoradionecrosis (p=0.39), post-RT stricture (p=0.24), or treatment-complication requiring surgery (p=0.50) (eTable 6).

Body-Mass Index association with survival and toxicity outcomes

We also stratified patients into overweight (BMI>25 kg/mm^2) and non-underweight (BMI≤25 kg/mm^2) groups based on World Health Organization (WHO) classification. OS was associated with underweight in univariable analysis (eTable 7; HR 1.95 [95% CI 1.19–3.19], p=0.008) but not in multivariable analysis (eTable 7; HR 1.53 [95% CI 0.92–2.52], p=0.10). Overweight-based model had higher Akaike's information criterion (AIC) and Bayesian information criterion (BIC) values than sarcopenia-based model (Table S8; AIC: 702.4 vs. 700.0; BIC: 725.3 vs. 722.9). PEG tube duration was associated with overweight in multivariable analysis (eTable 10; HR 1.52 [95% CI 1.14–2.04], p=0.004) and overweight-based model had lower AIC and BIC values (eTable 11; AIC: 2480.5 vs. 2482.3; BIC: 2502.6 vs. 2504.4).

HPV subgroup analysis

We performed a subgroup analysis for patients with HPV status (n=225). Sarcopenia was associated with both OS (eTable 13; HR 1.74 [95% CI 0.67-4.52], p=0.02) and PEG tube duration (eTable 14; HR 0.67 [95% CI 0.48-0.94], p=0.02) on univariable analysis. However, on multivariable analysis, OS was only associated with HPV status (eTable 13; HR 2.53 [95% CI 1.18-5.40], p=0.02), while PEG tube duration was only associated smoking history (eTable 14; HR 0.67 [95% CI 0.50-0.91], p=0.009).

Discussion

We utilized the pre-defined sex-specific cut-off values proposed by Prado et al.[8] to determine sarcopenia. We found female patients had significantly lower SMI values than males, consistent with previous studies. There is currently no consensus on the optimal method to define sarcopenia, and several other proposed thresholds exist. The end-to-end DL pipeline we developed for fully automated C3 segmentation allows for the efficient analysis of a large number of CT images. Traditional approaches involving manual or semi-automated C3 segmentation are laborious and require substantial expertise, making it challenging to analyze large datasets, particularly in multi-institutional studies. In the future, we aim to expand our study to include international multi-institutional patient cohorts to identify optimal cut-off values for sarcopenia through further analyses, such as receiver-operating characteristics and precision-recall analyses. We hope this will establish a more reliable association between sarcopenia and clinical risk factors for HNSCC patients.

eReferences

1. Wahid KA, Olson B, Jain R, et al. Muscle and adipose tissue segmentations at the third cervical vertebral level in patients with head and neck cancer. *Sci Data*. 2022;9(1):470. doi:10.1038/s41597-022-01587-w
2. Huang G, Liu Z, Van Der Maaten L, Weinberger KQ. Densely Connected Convolutional Networks. In: *2017 IEEE Conference on Computer Vision and Pattern Recognition (CVPR)*. ; 2017:2261-2269. doi:10.1109/CVPR.2017.243

3. Ronneberger O, Fischer P, Brox T. U-net: Convolutional networks for biomedical image segmentation. In: *Lecture Notes in Computer Science (Including Subseries Lecture Notes in Artificial Intelligence and Lecture Notes in Bioinformatics)*. Vol 9351. ; 2015. doi:10.1007/978-3-319-24574-4_28
4. Bridge CP, Rosenthal M, Wright B, et al. Fully-automated analysis of body composition from CT in cancer patients using convolutional neural networks. In: *Lecture Notes in Computer Science (Including Subseries Lecture Notes in Artificial Intelligence and Lecture Notes in Bioinformatics)*. Vol 11041 LNCS. ; 2018. doi:10.1007/978-3-030-01201-4_22
5. Swartz JE, Pothen AJ, Wegner I, et al. Feasibility of using head and neck CT imaging to assess skeletal muscle mass in head and neck cancer patients. *Oral Oncol*. 2016;62. doi:10.1016/j.oraloncology.2016.09.006
6. van Rijn-Dekker MI, van den Bosch L, van den Hoek JGM, et al. Impact of sarcopenia on survival and late toxicity in head and neck cancer patients treated with radiotherapy. *Radiotherapy and Oncology*. 2020;147. doi:10.1016/j.radonc.2020.03.014
7. Nguyen-Tan PF, Zhang Q, Ang KK, et al. Randomized phase III trial to test accelerated versus standard fractionation in combination with concurrent cisplatin for head and neck carcinomas in the radiation therapy oncology group 0129 trial: Long-term report of efficacy and toxicity. *Journal of Clinical Oncology*. 2014;32(34). doi:10.1200/JCO.2014.55.3925
8. Prado CM, Lieffers JR, McCargar LJ, et al. Prevalence and clinical implications of sarcopenic obesity in patients with solid tumours of the respiratory and gastrointestinal tracts: a population-based study. *Lancet Oncol*. 2008;9(7). doi:10.1016/S1470-2045(08)70153-0

eTables

eTable 1. CT Scanner Manufacturers and Models Used for Head and Neck Scans

Manufacturer	Model	Number
GE Medical Systems	LightSpeed RT	882
GE Medical Systems	LightSpeed QX/i	11
GE Medical Systems	Discovery CT590 RT	2
SIEMENS	SOMATOM Confidence	4

eTable 2. Head and Neck CT Scan Characteristic Deviation Table

Scan Characteristic	Mean	Median	Mode	Range (min – max)	Standard Deviation
Pixel Size (mm)	0.89	0.98	0.98	(0.34 – 2.34)	0.33
Slice Thickness (mm)	2.48	2.50	2.50	(1.25 – 3.0)	0.15
Tube Voltage (kVp)	120.0	120.0	120.0	(120.0 – 140.0)	1.29

© 2023 Ye Z et al. *JAMA Network Open.*

eTable 3. Summary on Failing Cases on External Test Set

Failing Causes	Failing Numbers (n = 23)
Missing sternocleidomastoid muscle (SCM)	9 (39.1%)
Lymph node included	6 (26.1%)
Post neck muscle missing	3 (13%)
Anterior deep muscle missing	3 (13%)
Submental muscle included	1 (4.4%)
Other muscle included	1 (4.4%)

eTable 4. U-Net Segmentation Model Performance

	DSC	Precision	Recall	ICC
Validation Set<br>(n = 93)	0.90<br>(0.90 – 0.91)	0.97<br>(0.96 – 0.97)	0.84<br>(0.84 - 0.85)	0.99<br>(0.98 – 0.99)
Internal Test Set<br>(n = 48)	0.90<br>(0.89 – 0.91)	0.97<br>(0.95 – 0.97)	0.85<br>(0.83 – 0.85)	0.96<br>(0.94 – 0.98)

eTable 5. Patient Characteristics for Nonsarcopenic and Sarcopenic Groups

Patient Cohort<br>(n = 342)	Sarcopenic<br>(n = 261)	Non-Sarcopenic<br>(n = 81)	*p*-value
**Gender**			0.99*
Male	216 (82.76%)	67 (82.72%)	
Female	45 (17.24%)	14 (17.28%)	
**Smoker**			0.53^+
Former	126(48.28%)	42 (51.85%)	
Smoking at initial consult	40 (15.33%)	9 (11.11%)	
Never	94 (36.02%)	29 (35.80%)	
Unspecified/Unknown	1 (0.38%)	1 (1.23%)	
**Hospital during RT**			0.59^+
Yes	62 (23.75%)	19 (23.46%)	
No	198 (75.86%)	61 (75.31%)	
Unspecified/Unknown	1 (0.38%)	1 (1.23%)	
**PEG Tube Insert**			0.14^+
Yes	237 (90.80%)	68 (83.95%)	
No	23 (8.81%)	12 (14.81%)	
Unspecified/Unknown	1 (0.38%)	1 (1.23%)	

© 2023 Ye Z et al. *JAMA Network Open.*

**T-Stage**			0.18*
T1	50 (19.16%)	24 (29.63%)	
T2	100 (38.31%)	31 (38.27%)	
T3	75 (28.74%)	18 (22.22%)	
T4	36 (13.79%)	8 (9.88%)	
Unspecified/Unknown	0	0	
**N-Stage**			0.27*
N0	52 (19.92%)	17 (20.99%)	
N1	28 (10.73%)	10 (12.35%)	
N2	167 (63.98%)	45 (55.56%)	
N3	14 (5.36%)	9 (11.11%)	
Unspecified/Unknown	0	0	
**HPV Status**			0.12*
+	138 (52.87%)	49 (60.49%)	
-	34 (13.03%)	4 (4.94%)	
Unspecified/Unknown	89 (34.10%)	28 (34.57%)	

Note: Chi-squared test for the independence (∗) or Fisher's exact test (+) were used for group comparisons between non-sarcopenic and sarcopenic groups for each gender. Fisher's exact test was used if the expected values of Chi-squared test were smaller than 5. SMI: skeletal muscle index. HPV: Human papillomavirus. RT: radiotherapy. PEG: percutaneous endoscopic gastrostomy.

eTable 6. Univariable Analysis for the Association of sarcopenia With Various Toxicity End Points

**Toxicity**	**No Sarcopenia**	**Sarcopenia**	**OR (95% CI)**	***p*-value**
PEG inserted at diagnosis	84%	91%	1.81 (0.86 - 3.84)	0.12
PEG removal	77%	75%	0.46 (0.19 - 1.14)	0.09
PEG at last follow up	**10%**	**18%**	**2.25 (1.02 - 4.99)**	**0.05**
Hospitalization during RT	23%	24%	1.01 (0.56 - 1.81)	0.97
Hospitalization < 3 months after RT	6%	13%	2.18 (0.82 - 5.79)	0.12
Osteoradionecrosis	2%	1%	0.45 (0.07 - 2.77)	0.39
Post-RT stricture	7%	12%	1.73 (0.70 - 4.30)	0.24
Complication from treatment requiring surgery	0.6%	3.5%	0.86 (0.40 – 1.85)	0.70

OR: odds ration; RT: radiotherapy; PEG: percutaneous endoscopic gastrostomy. Bold text indicated statistical significance.

© 2023 Ye Z et al. *JAMA Network Open.*

eTable 7. Baseline Univariable and Multivariable Analyses for Overall Survival With Underweight

	Univariable Analysis		Multivariable Analysis	
	**HR (95% CI)**	**p-value**	**HR (95% CI)**	**p-value**
**Underweight**				
No (BMI ≥ 18.5)	**Ref**		Ref	
Yes (BMI < 18.5)	**4.70 (1.70 - 12.9)**	**0.003**	2.01 (0.71 - 5.74)	0.19
**Age**				
< 65	**Ref**		Ref	
≥ 65	**1.93 (1.20 - 3.10)**	**0.007**	1.20 (0.72 - 2.03)	0.48
**Smoking History**				
< 10py	**Ref**		Ref	
≥ 10py	**2.00 (1.23 - 3.25)**	**0.005**	1.11 (0.65 - 1.92)	0.70
**ACE-27 score**				
0-1	**Ref**		**Ref**	
2-3	**2.24 (1.39 - 3.62)**	**0.001**	**1.86 (1.11 - 3.14)**	**0.02**
**Tumor Site**				
Oropharynx primary	**Ref**		**Ref**	
Non-oropharynx	**3.92 (2.45 - 6.25)**	**< 0.001**	**3.20 (1.91 - 5.38)**	**< 0.001**
**T-Stage**				
T1-2	**Ref**		**Ref**	
T3-4	**2.36 (1.47 - 3.77)**	**< 0.001**	**2.46 (1.50 - 4.03)**	**< 0.001**
**N-Stage**				
N0-1	Ref			
N2-3	0.88 (0.54 - 1.43)	0.60		
**AJCC 7th Stage**				
Stage 1-2	Ref			
Stage 3-4	1.31 (0.69 - 2.49)	0.41		

HR: hazard ratio; CI: confidence interval; Ref: reference. Bold text indicated statistical significance. Bold text indicated statistical significance.

eTable 8. Baseline Univariable and Multivariable Analyses for Overall Survival With Overweight

	Univariable Analysis		Multivariable Analysis	
	**HR (95% CI)**	**p-value**	**HR (95% CI)**	**p-value**
**Overweight**				
No (BMI ≤ 25)	**Ref**		Ref	
Yes (BMI > 25)	**1.95 (1.19 - 3.19)**	**0.008**	1.53 (0.92 - 2.52)	0.10
**Age**				
< 65	**Ref**		Ref	
≥ 65	**1.93 (1.20 - 3.10)**	**0.007**	1.20 (0.71 - 2.02)	0.51
**Smoking History**				
<10py	**Ref**		Ref	
≥10py	**2.00 (1.23 - 3.25)**	**0.005**	1.12 (0.66 - 1.92)	0.67

© 2023 Ye Z et al. *JAMA Network Open.*

**ACE-27 score**				
0-1	**Ref**		**Ref**	
2-3	**2.24 (1.39 - 3.62)**	**0.001**	**1.82 (1.09 - 3.06)**	**0.02**
**Tumor Site**				
Oropharynx primary	**Ref**		**Ref**	
Non-oropharynx	**3.92 (2.45 - 6.25)**	**< 0.001**	**3.18 (1.89 - 5.34)**	**< 0.001**
**T-Stage**				
T1-2	**Ref**		**Ref**	
T3-4	**2.36 (1.47 - 3.77)**	**< 0.001**	**2.52 (1.55 - 4.13)**	**< 0.001**
**N-Stage**				
N0-1	Ref			
N2-3	0.88 (0.54 - 1.43)	0.60		
**AJCC 7th Stage**				
Stage 1-2	Ref			
Stage 3-4	1.31 (0.69 - 2.49)	0.41		

HR: hazard ratio; CI: confidence interval; Ref: reference. Bold text indicated statistical significance.

eTable 9. AIC and BIC for Overall Survival Model

Model	N	ll (Null)	ll (Model)	DF	AIC	BIC
Sarcopenia	338	-371.3	-344.0	6	700.0	722.9
Underweight (BMI < 18.5)	338	-371.3	-345.8	6	703.6	726.5
Overweight (BMI > 25)	338	-371.3	-345.2	6	702.4	725.3

N = number of observations; DF: degree of freedom; AIC: Akaike's information criterion; BIC: Bayesian information criterion.

eTable 10. Multivariable Analyses for PEG Tube Duration With Underweight

	**Multivariable Analysis**	
	**HR (95% CI)**	**p-value**
**Underweight**		
No (BMI ≥ 18.5)	Ref	
Yes (BMI < 18.5)	0.97 (0.36 – 2.64)	0.95
**Age**		
< 65	**Ref**	
≥ 65	**0.72 (0.53 – 0.97)**	**0.03**
**Smoking History**		
< 10py	**Ref**	
≥ 10py	**0.66 (0.51 – 0.86)**	**0.002**
**ACE-27 score**		
0-1	Ref	
2-3	0.74 (0.55 – 1.01)	0.06

© 2023 Ye Z et al. *JAMA Network Open.*

**Tumor Site**		
Oropharynx primary	Ref	
Non-oropharynx	1.23 (0.86 – 1.76)	0.26
**T-Stage**		
T1-2	**Ref**	
T3-4	**0.76 (0.59 – 0.98)**	**0.03**

HR: hazard ratio; CI: confidence interval; Ref: reference. Bold text indicated statistical significance. Bold text indicated statistical significance.

eTable 11. Univariable and Multivariable Analyses for PEG Tube Duration With Overweight

	**Multivariable Analysis**	
	**HR (95% CI)**	**p-value**
**Overweight**		
No (BMI ≤ 25)	**Ref**	
Yes (BMI > 25)	**1.52 (1.14 – 2.04)**	**0.004**
**Age**		
< 65	**Ref**	
≥ 65	**0.70 (0.52 – 0.95)**	**0.02**
**Smoking History**		
<10py	**Ref**	
≥10py	**0.66 (0.51 – 0.86)**	**0.002**
**ACE-27 score**		
0-1	Ref	
2-3	0.77 (0.57 – 1.05)	0.10
**Tumor Site**		
Oropharynx primary	Ref	
Non-oropharynx	1.20 (0.84 – 1.72)	0.31
**T-Stage**		
T1-2	Ref	
T3-4	0.79 (0.61 – 1.01)	0.07

HR: hazard ratio; CI: confidence interval; Ref: reference. Bold text indicated statistical significance. Bold text indicated statistical significance.

eTable 12. AIC and BIC for PEG Tube Duration Model

**Model**	**N**	**ll (Null)**	**ll (Model)**	**DF**	**AIC**	**BIC**
Sarcopenia	294	-1255.07	-1235.17	6	2482.34	2504.44
Underweight (BMI < 18.5)	294	-1255.07	-1238.65	6	2489.29	2511.40
Overweight (BMI > 25)	294	-1255.07	-1234.26	6	2480.52	2502.62

N: number of observations; DF: degree of freedom; AIC: Akaike's information criterion; BIC: Bayesian information criterion.

eTable 13. Univariable and Multivariable Analyses for Overall Survival With Available HPV Status (n = 225)

	**Univariable Analysis**		**Multivariable Analysis**	
	**HR (95% CI)**	**p-value**	**HR (95% CI)**	**p-value**
**Sarcopenia**				
No	**Ref**			
Yes	**1.74 (0.67-4.52)**	**0.02**		
**Age**				
<65	**Ref**			
≥65	**1.17 (0.48-2.85)**	**0.02**		
**HPV Status**				
Positive	**Ref**		Ref	
Negative	**2.69 (1.27-5.72)**	**0.005**	**2.53 (1.18-5.40)**	**0.02**
**Smoking History**				
<10py	Ref			
≥10py	**1.70 (0.84-3.45)**	**0.002**		
**ACE-27 score**				
0-1	Ref			
2-3	1.84 (0.87-3.91)	0.05		
**Tumor Site**				
Oropharynx primary	Ref			
Non-oropharynx	2.23 (0.68-7.35)	0.42		
**T-Stage**				
T1-2	Ref			
T3-4	1.78 (0.88-3.59)	0.11		
**N-Stage**				
N0-1	Ref			
N2-3	0.77 (0.34-1.71)	0.28		
**AJCC 7th Stage**				
Stage 1-2	**Ref**			
Stage 3-4	3.13 (1.10-8.96)	0.19	2.72 (0.94-7.86)	0.06

HR: hazard ratio; CI: confidence interval; Ref: reference.

© 2023 Ye Z et al. *JAMA Network Open.*

eTable 14. Univariable and Multivariable Analyses for PEG Tube Duration With Available HPV Status (n = 225)

	**Univariable Analysis**		**Multivariable Analysis**	
	**HR (95% CI)**	**p-value**	**HR (95% CI)**	**p-value**
**Sarcopenia**				
No	**Ref**		Ref	
Yes	**0.67 (0.48 - 0.94)**	**0.02**	0.77 (0.54 - 1.09)	0.14
**Age**				
<65	**Ref**		Ref	
≥65	**0.64 (0.45 - 0.92)**	**0.02**	0.74 (0.50 - 1.07)	0.11
**HPV Status**				
Positive	**Ref**		Ref	
Negative	**0.57 (0.39 - 0.84)**	**0.005**	0.68 (0.46 - 1.02)	0.06
**Smoking History**				
<10py	Ref		**Ref**	
≥10py	**0.63 (0.47 - 0.85)**	**0.002**	**0.67 (0.50 - 0.91)**	**0.009**
**ACE-27 score**				
0-1	Ref			
2-3	0.70 (0.48 - 1.00)	0.05		
**Tumor Site**				
Oropharynx primary	Ref			
Non-oropharynx	0.76 (0.39 - 1.49)	0.42		
**T-Stage**				
T1-2	Ref			
T3-4	0.79 (0.59 - 1.05)	0.11		
**N-Stage**				
N0-1	Ref			
N2-3	1.21 (0.86 - 1.71)	0.28		
**AJCC 7th Stage**				
Stage 1-2	**Ref**			
Stage 3-4	0.51 (0.19 - 1.39)	0.19		

PEG tube duration was defined as the time from insertion of PEG tube to removal of PEG tube (i.e. HR < 1 represents longer time to removal or greater PEG tube duration). HR: hazard ratio; CI: confidence interval; Ref: reference. PEG: percutaneous endoscopic gastrostomy. Bold text indicated statistical significance.

© 2023 Ye Z et al. *JAMA Network Open.*

eTable 15. Fairness Assessment of Deep Learning Pipeline

Expert Clinicians	Clinical Acceptability	sex		Age		Smoking	
		Female	Male	< 65	≥ 65	Never	Current/Former
Reviewer 1	unacceptable	2 (3.2%)	10 (3.2%)	7 (1.9%)	5 (4.9%)	3 (2.7%)	7 (2.9%)
	acceptable	61 (96.8%)	304 (96.8%)	269 (98.1%)	97 (95.1%)	110 (97.3%)	236 (97.1%)
Reviewer 2	unacceptable	5 (7.9%)	14 (4.5%)	15 (5.4%)	4 (3.9%)	7 (6.2%)	10 (4.1%)
	acceptable	58 (92.1%)	300 (95.5%)	261 (94.6%)	98 (96.1%)	106 (93.8%)	233 (95.9%)

© 2023 Ye Z et al. *JAMA Network Open.*

eFigures

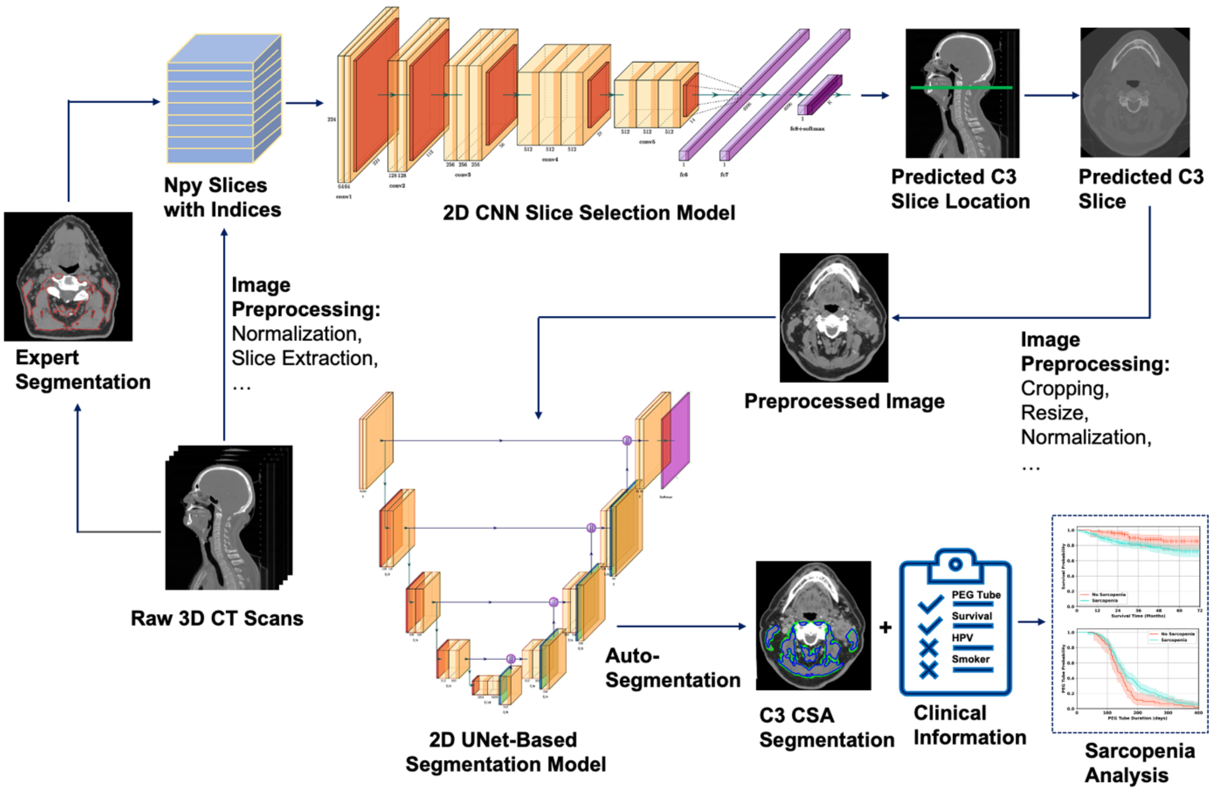

eFigure 1. Workflow of the Fully Automated Deep Learning Pipeline for Accurate C3 Segmentation. The 3-dimensional (3D) CT scans were first normalized and converted to 2-dimensional (2D) Numpy files with corresponding slice indices as inputs for slice selection model. The DenseNet regression model performed predictions on each individual axial slice, and then processed the results to determine the target C3 slice. The input to the model is a series of CT slices, and it learns to predict a single continuous value that represents the difference in position (z-offset) of the slice from the target C3 slice. Then the C3 slice was extracted from 3D CT scan and underwent a series of preprocessing steps including cropping, resizing and normalization. Subsequently the preprocessed C3 slice was fed into the U-Net segmentation model to segment the C3 SM and calculate the cross-sectional areas at the C3 level. The L3-SMI was derived to perform a series of predictive analyses. BWH: Brigham and Women's Hospital. DSC: dice similarity coefficient. CSA: C3 cross-sectional area; SMA: L3 skeletal muscle cross-sectional area. SMI: skeletal muscle index.

© 2023 Ye Z et al. *JAMA Network Open.*

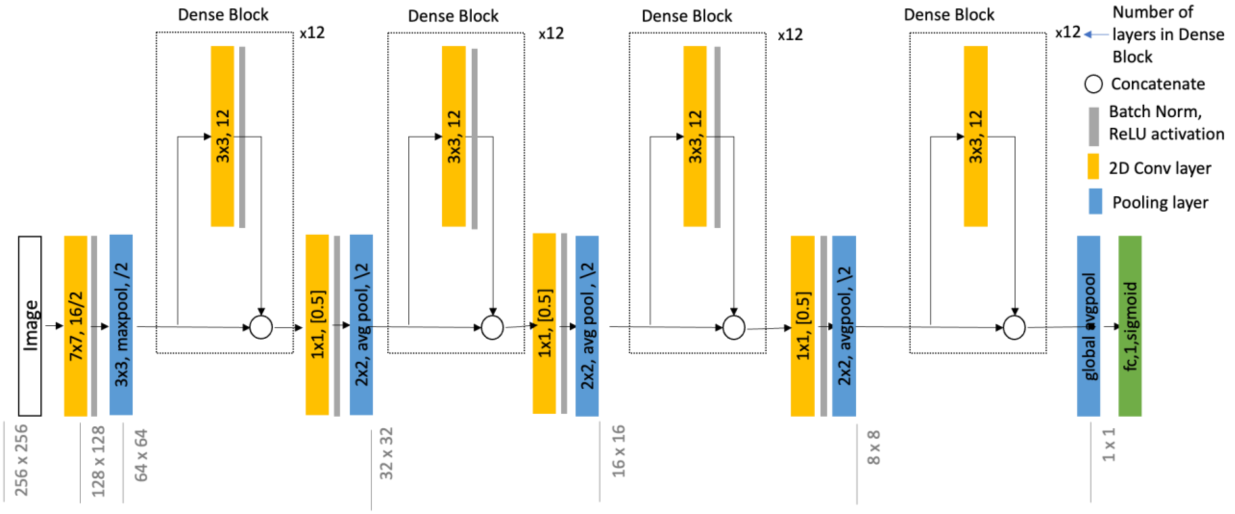

eFigure 2. Model Architecture for 2D DenseNet-Based Slice Selection Model, Adapted DenseNet CNN Architecture. Yellow blocks indicate 2D convolutional layers described by their kernel dimensions and number of output features. "x12" indicates 12 layers within each Dense Block. Gray blocks indicate batch normalization layer followed by ReLu activation. Blue blocks represent pooling layers. "/2" indicates that the conv/pool layer has stride 2, otherwise the stride is 1. In the DenseNet transition blocks '[0.5]' indicates the number of output features is half the number of input features (a compression factor of 0.5).

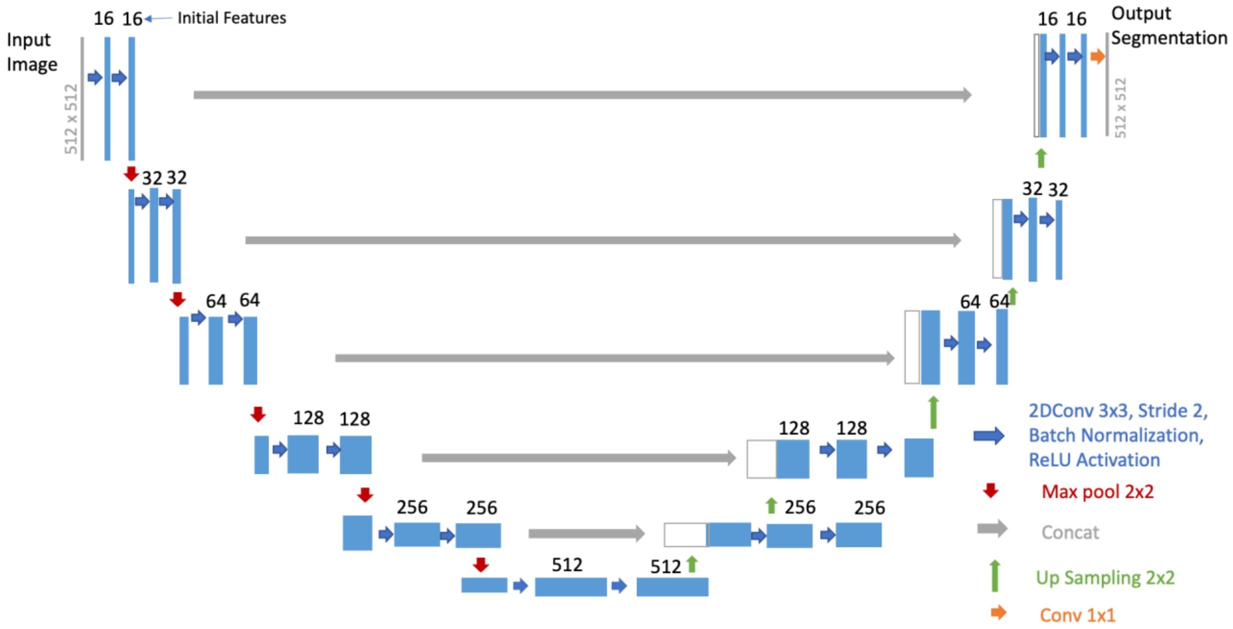

eFigure 3. Model Architecture for 2D U-Net–Based Segmentation Model. The number of features specified above each block.

© 2023 Ye Z et al. *JAMA Network Open.*

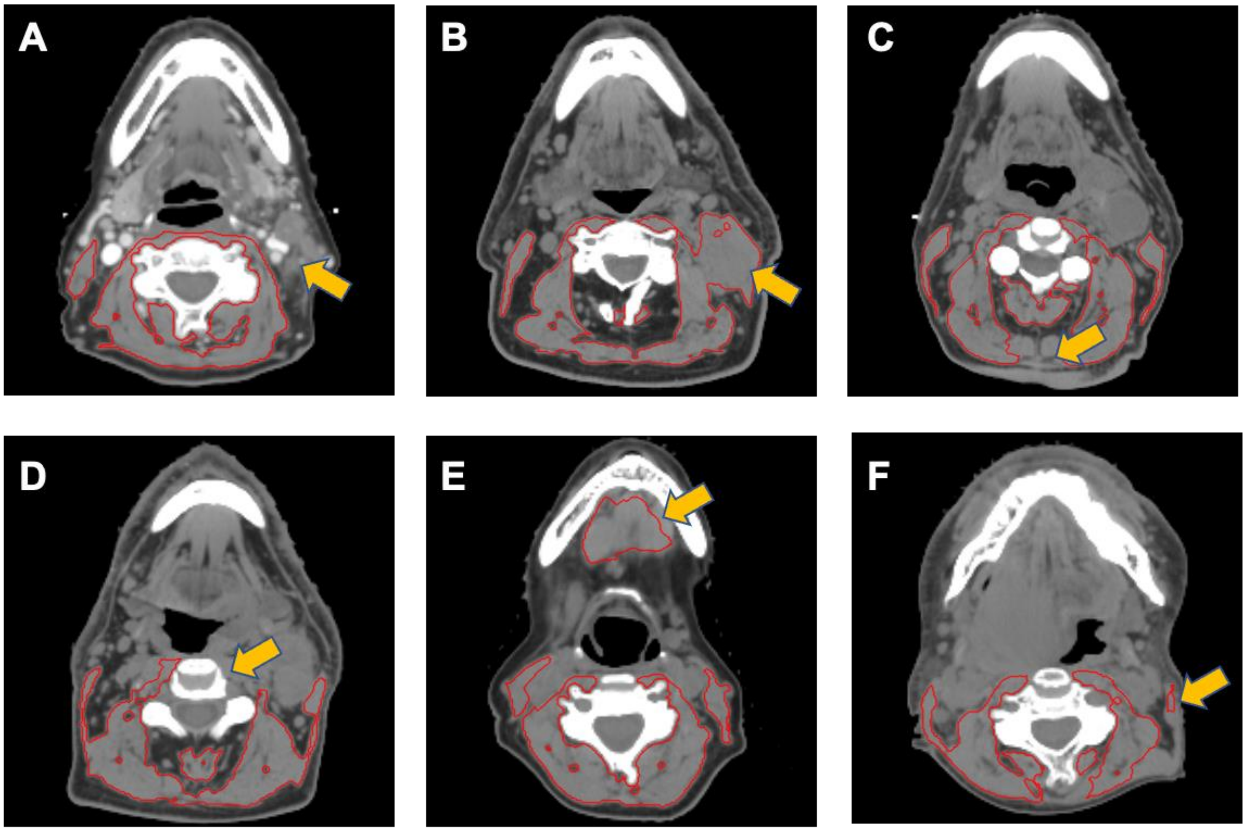

eFigure 4. Representative Failing Cases With Different Failing Causes. (A) Left sternocleidomastoid muscle (SCM) was missing; (B) Lymph node was included; (C) Post neck muscle was missing. (D) Anterior deep muscle was missing. (E) Submental muscle was included. (F) Other muscle was included.

© 2023 Ye Z et al. *JAMA Network Open.*

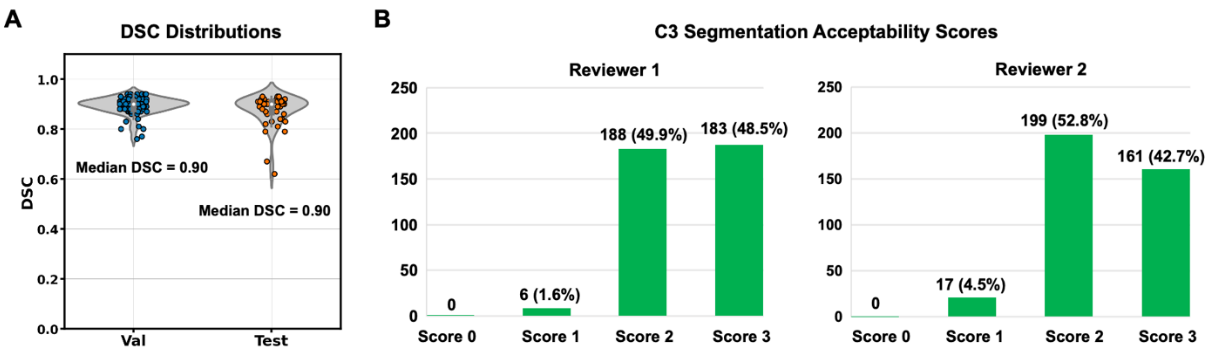

eFigure 5. C3 Segmentation Showed Excellent Performance in Both DSC Evaluation and Clinical Acceptability Evaluation. (A) DSC distributions were shown for validation and internal test sets. (B) C3 segmentations predicted by model were individually reviewed by two experienced board-certified radiation oncologists with Likert scales of 0 - 3: 0: the model selected an incorrect axial slice for segmentation that does not correspond to the C3-vertebral body; 1: the segmentation is unacceptable (defined as an estimated >5% muscle volume discrepancy compared to an expert segmentation); 2: the segmentation is clinically acceptable, though compared to expert segmentation would result in a small volume discrepancy ≤5%; and 3: segmentation is acceptable with no difference from expert segmentation. IQR: inter quantile range. DSC: Dice similarity coefficient.

© 2023 Ye Z et al. *JAMA Network Open.*

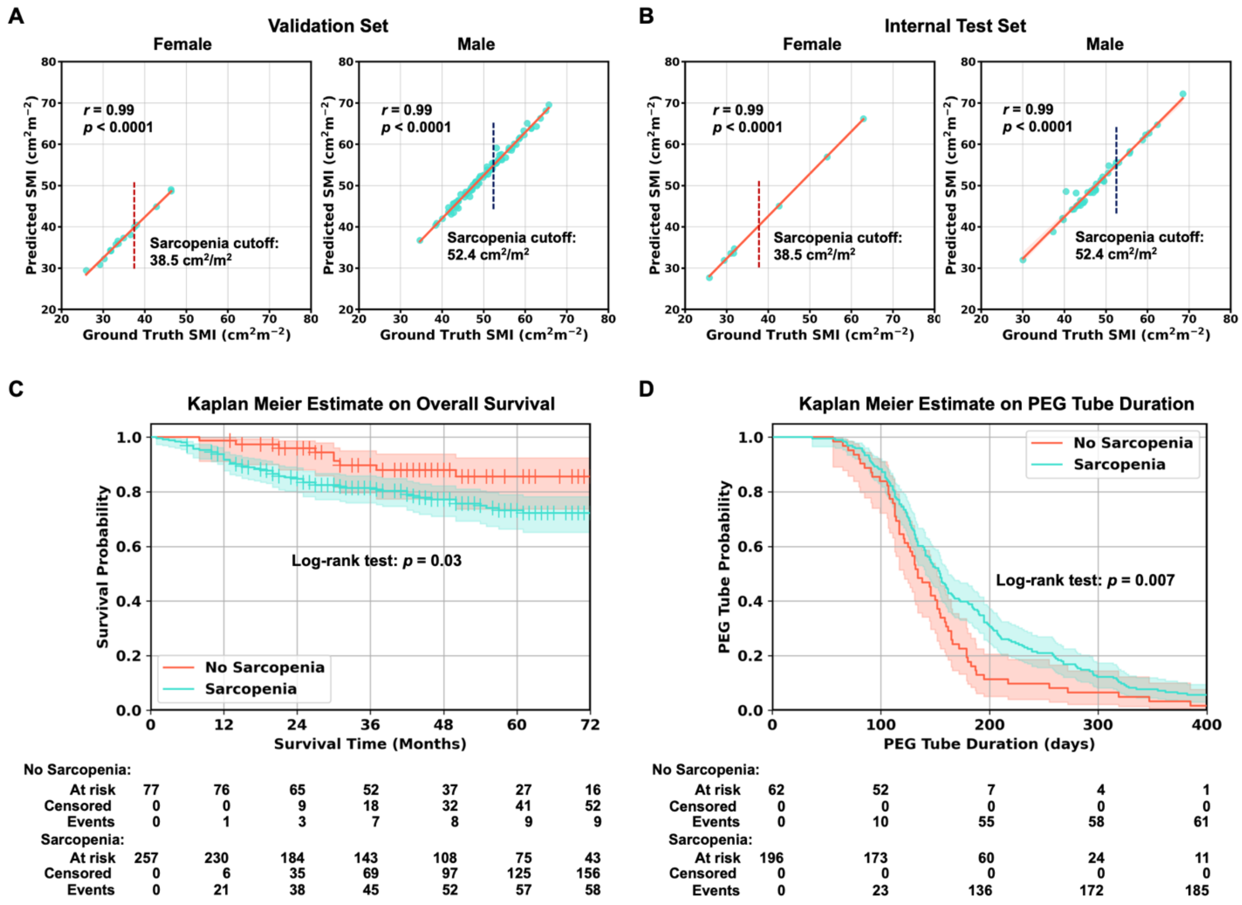

eFigure 6. Scatter Plots of the Skeletal Muscle Index (SMI) Values and Kaplan-Meier Survival Estimates. Scatter plots of the skeletal muscle index (SMI) values determined for validation set (A) and internal test set (B) patients (stratified by sex) using the ground-truth manual segmentation (x-axis) and model predicted segmentations (y-axis). Pearson's correlations showed all model-predicted values and ground truth values were significantly correlated ($p < 0.0001$). SMI thresholds of 52.4 cm^2/m^2 for males and 38.5 cm^2/m^2 for females were adopted to stratify patients into sarcopenia and non-sarcopenia groups (A&B, dash lines). Kaplan-Meier curves show significant differences in both overall survival time (C; Log-rank test $p = 0.03$) and PEG tube duration (D; Log-rank test $p = 0.007$) between sarcopenia patients and no sarcopenia patients in the external test set.

© 2023 Ye Z et al. *JAMA Network Open.*
