# Supplementary material for: Development and Validation of an Automated Image-Based Deep Learning Platform for Sarcopenia Assessment in Head and Neck Cancer
Source: JAMA Netw Open. 2023 Aug 10;6(8):e2328280. doi: 10.1001/jamanetworkopen.2023.28280 (PMC10415962; doi:10.1001/jamanetworkopen.2023.28280)
Supplement: Supplement 2. — Data Sharing Statement [file jamanetwopen-e2328280-s002.pdf]

Data Sharing Statement

Ye. Development and Validation of an Automated Image-Based Deep Learning Platform for Sarcopenia Assessment in Head and Neck Cancer. *JAMA Netw Open*. Published August 10, 2023. doi:10.1001/jamanetworkopen.2023.28280

Data
Data available: Yes
Data types: Deidentified participant data
How to access data: Benjamin_Kann@DFCI.HARVARD.EDU
When available: With publication

Supporting Documents
Document types: Statistical/analytic code
How to access documents: https://github.com/xmuyzz/C3-Segmentation
When available: With publication

Additional Information
Who can access the data: anyone requesting the data
Types of analyses: for a specified purpose
Mechanisms of data availability: with investigator support
